# Brain Metastasis Occurring a Decade After Prostate Cancer Diagnosis: A Case Report

**DOI:** 10.7759/cureus.109715

**Published:** 2026-05-27

**Authors:** Arun Arumugam, Ronald Meira Castro Trindade, Amara Hendricks, Matthew R Powell, Juhi Bansal

**Affiliations:** 1 Radiology, Augusta University Medical College of Georgia, Augusta, USA; 2 Pathology, Augusta University Medical College of Georgia, Augusta, USA

**Keywords:** brain cancer, metastasis, neuroradiology, prostate cancer, radiology, urology

## Abstract

Brain metastases are rare, yet severe manifestations of prostate cancer that present with symptoms such as severe headaches, seizures, confusion, and memory deficits. Here we describe a unique case of a 77-year-old patient with a history of Gleason 3+4 prostate cancer nearly a decade prior that was determined to have brain metastasis due to similar symptoms. Utilization of imaging tools, including non-contrast CT scans, susceptibility-weighted imaging, and MRIs, helped with assessment of the lesion, and histopathological analysis guided treatment. This case highlights the importance of considering delayed brain metastasis in patients with prior prostate cancer and new neurological deficits. Prompt radiological evaluation and pathologic confirmation are essential for accurate diagnosis and management.

## Introduction

Prostate cancer is the most commonly diagnosed malignancy and the second-highest cause of cancer-associated death in men due to its tendency to metastasize to other organs [1, 2]. Metastatic spread commonly involves the axial skeleton in up to 80% of cases, with the frequent spread to the spine, pelvis, and ribs [3, 4]. The mechanism of osseous involvement is believed to occur through hematogenous spread through Batson's venous plexus in addition to the interaction of the tumor cells with the bone microenvironment [3].

Prostate cancer has additionally been shown to metastasize atypically to organs such as the breasts, bladder, and kidneys [5]. One rare form of prostate cancer metastasis is to the brain of affected men, with an occurrence rate ranging from only 0.6% to 2.0% [6-8]. Brain metastasis is typically associated with advanced-stage malignancy, aggressive variants, and widespread metastasis [9]. The rarity of such an occurrence has led to minimal current studies and a potential lack of awareness. Therefore, physicians often may not consider its viability, leading to potential misdiagnoses and a delay of treatment [6].

Certain manifestations of brain metastasis from prostate cancer include headaches and diplopia due to metastasis increasing intracranial pressure and involving cranial nerves in control of eye movements. These findings are highly nonspecific and require further investigation into a patient’s past medical history to derive the correct diagnosis. In addition, some common manifestations of brain metastases from prostate cancer are short-term memory deficits and delirium, which can make it difficult for the physician to pinpoint a cause due to difficulty in communicating with the patient [5]. In this report, we describe a case of a patient with brain metastasis from prostate cancer, the associated symptoms, and how the cause was determined to be due to prostate metastasis through multimodal imaging and histopathological analysis.

## Case presentation

A 77-year-old male patient presented to the hospital with complaints of headaches, diplopia, dysphagia, and gait instability that progressively worsened over a month. In addition, he reported significant difficulty in controlling his lower extremities but denied any similar struggles with control of his upper extremities. On physical examination, he had a normal cranial nerve examination except for a decreased gag reflex and loss of sensation of the left V2 & V3 distributions, hyperreflexia in bilateral lower extremities, 5/5 muscle strength throughout, no dysmetria or drift, and difficulty with heel-to-toe testing. The patient did not experience diplopia at this time. Nine years prior, he was diagnosed with T2a adenocarcinoma of the prostate with an elevated Gleason score of 3 + 4 = 7 and relative perineural invasion that was thereafter treated with radiation therapy with improvement. Computed tomography (CT), magnetic resonance imaging (MRI), and susceptibility-weighted imaging (SWI) scans of the head were taken two days after to identify a potential diagnosis (Figures 1-4).

**Figure 1:**
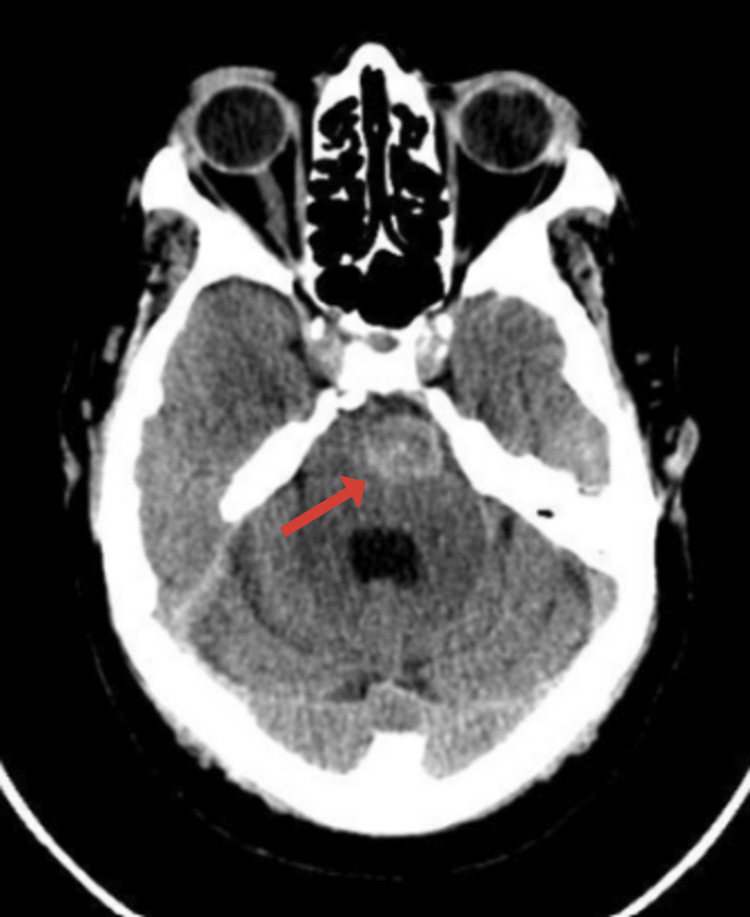
Non-contrast CT of the head Axial non-contrast CT image demonstrating a hyperdense lesion centered in the left pons, associated with surrounding hypodensity consistent with vasogenic edema.

**Figure 2:**
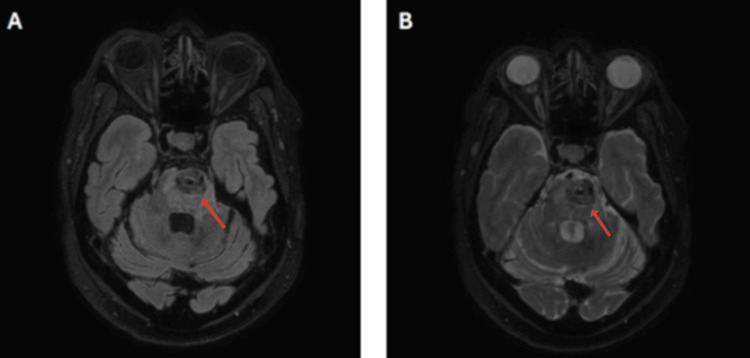
Fluid-attenuated inversion recovery (FLAIR) and T2-weighted MRI of the head A. Axial FLAIR MRI demonstrating a target-like signal distribution, characterized by a more hypointense central nodular area encircled by a peripheral hypointense halo. B. T2-weighted MRI highlighting the heterogenous hypointense lesion centered in the left pons.

**Figure 3:**
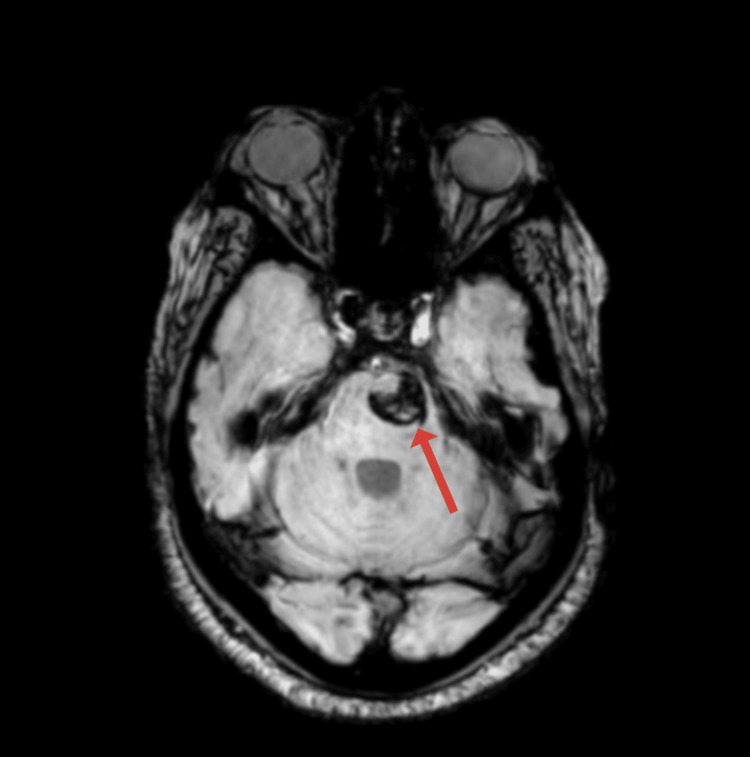
Axial susceptibility-weighted imaging (SWI) of the head Axial SWI demonstrating prominent blooming artifacts within​ the heterogeneous pontine lesion.

**Figure 4:**
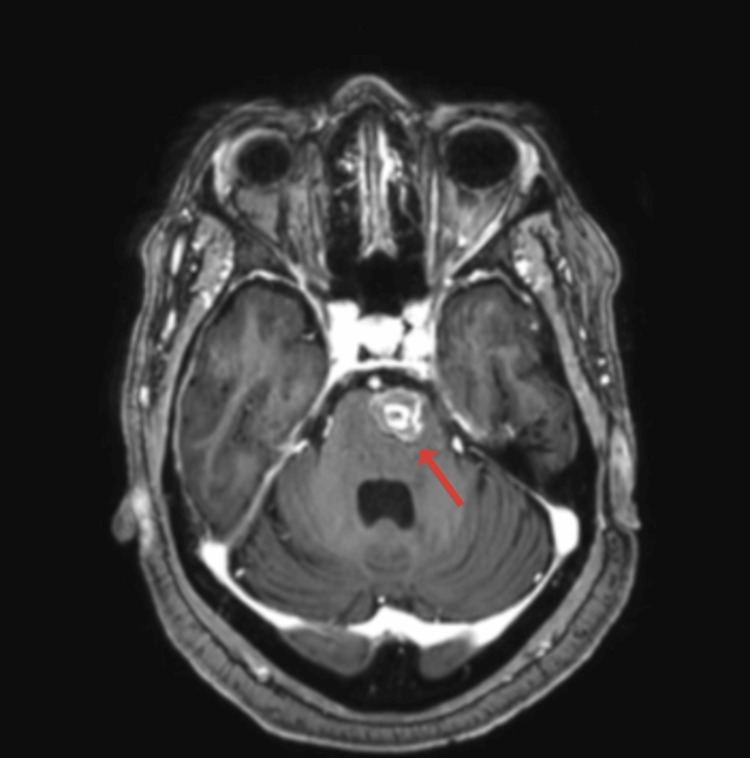
Axial post-contrast T1-weighted MRI of the head Axial post-contrast T1-weighted MRI demonstrating a target-like appearance, with a centrally enhancing nodular core surrounded by a peripheral ring of enhancement, consistent with its previously noted internal architecture.

After identification of a pontine lesion through imaging, CT scans of the abdomen, pelvis, and chest were then taken to visualize regions of potential metastasis. This imaging identified a 35 x 16 x 15 mm pulmonary mass that helped narrow down the suspicion that the pontine lesion may be due to metastasis. Vasogenic edema noted intracranially was treated with a lumbar drain to remove the excess fluid, and an endoscopic endonasal biopsy and resection of the lesion, through a transclival approach, was undertaken by neurosurgery and otolaryngology (Figure 5).

**Figure 5:**
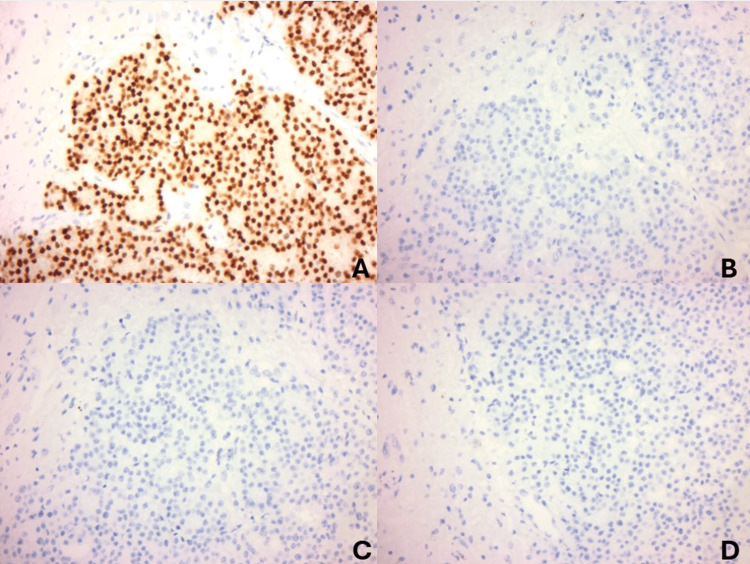
Histopathological analysis of the biopsy. A. Immunohistochemistry for NKX3.1 demonstrates strong, diffuse nuclear positivity in malignant epithelial cells, supporting prostatic origin. The surrounding brainstem parenchyma is negative, serving as an internal negative control (200X). B. TTF1 immunochemistry stain showing negative nuclear staining in the infiltrating malignant epithelial cells, arguing against pulmonary or thyroid origin in this morphologic and immunophenotypic context (200X). C & D. Immunochemistry for CK7 and CK20, respectively, showing negative staining in the infiltrating malignant epithelial cells, a profile compatible with metastatic prostatic adenocarcinoma in this morphologic and immunophenotypic context (200X).

The resulting biopsy distinguished that the pontine lesion originated from metastatic prostatic adenocarcinoma, the same diagnosis the patient was given nearly a decade before. Resection of the lesion was undertaken, and the patient was hospitalized postoperatively. He remained neurologically stable with no signs of headache or diplopia and was instructed to follow up with hematology/oncology after discharge. The patient’s most recent CT scan, one year postoperatively, reported no significant masses in the brain.

## Discussion

Metastasis of prostate adenocarcinoma to the brain is a rare, yet severe, event that occurs in less than 2% of cases and can present as nonspecific neurological symptoms in a patient [6]. Utilization of imaging can help with the assessment of lesions and discerning between imaging characteristics of other central nervous system (CNS) pathologies due to the critical location involved. In this case, the patient developed worsening neurological symptoms close to a decade after his initial prostate cancer diagnosis. A non-contrast CT of his brain demonstrated a hyperdense lesion in the left pons, and a subsequent MRI taken revealed a T1-weighted hypointense, T2-weighted/fluid-attenuated inversion recovery (FLAIR) hyperintense lesion with heterogeneous enhancement on post-contrast images, and SWI demonstrated marked intralesional blooming artifacts. While nonspecific, similar findings have been reported in previous cases of brain metastases from prostate adenocarcinoma and were important to consider when formulating a differential diagnosis [10]. While advanced sequences such as perfusion and MR spectroscopy can help in tumor evaluation, conventional and susceptibility-based sequences were sufficient to raise concern for metastatic disease in this scenario [11].

The differential diagnosis of a solitary brainstem lesion on imaging of an adult patient includes various considerations. Brainstem gliomas, although more common in pediatric populations, can present in adults as a solitary brainstem lesion and similarly present with diffuse T2 hyperintensity. But these lesions will have minimal to no enhancement, which was not seen in our case of metastasis [12]. Primary CNS lymphoma, another diagnosis considered due to the overlap of imaging characteristics, frequently shows homogeneous contrast enhancement and diffusion restriction, but calcification and hemorrhage are uncommon [13]. Cavernous malformations can similarly present as a lesion with blooming artifacts on SWI but usually additionally demonstrate a popcorn-like appearance with a lack of associated contrast enhancement [14]. Due to the variance of diagnoses that are possible, utilization of more than one modality of imaging is imperative to distinguish among pathologies.

Alongside the utilization of imaging, histopathological analysis of the biopsy ultimately confirmed the suspected diagnosis of metastatic prostatic adenocarcinoma for this patient. Strong diffuse nuclear positivity for NKX3.1 within the malignant epithelial cells helped support a prostatic origin of the tumor, and additional staining displayed negative TTF1 expression, helping to rule out a primary pulmonary or thyroid malignancy. Cases such as these highlight the need for clinicians to consider delayed-onset brain metastasis in patients with a history of prostate cancer, particularly when they present with neurological symptoms such as headaches, diplopia, and gait instability. Combined imaging modalities, such as SWI, play an important role in the detection and interpretation of such lesions, especially in surgically challenging locations such as the pons, and a biopsy can help definitively confirm the accurate diagnosis.

## Conclusions

Although highly uncommon, metastasis of prostate cancer to the brain is a rare but clinically significant occurrence that physicians must be aware of. Identification of symptoms such as delirium, memory deficits, and diplopia through thorough neurological examinations on patients with a history of prostate cancer can help facilitate earlier recognition of brain involvement. Once suspected, initial imaging can help interpret lesions suspicious for metastasis, while pathologic analysis can help confirm the diagnosis and guide subsequent management appropriately. Afterwards, surgical intervention, including drainage and resection, may help reduce mass effect, improve neurological symptoms, and increase quality of life in selected patients.
